# Rapid Mass Movement of Chloroplasts during Segment Formation of the Calcifying Siphonalean Green Alga, *Halimeda macroloba*

**DOI:** 10.1371/journal.pone.0020841

**Published:** 2011-07-05

**Authors:** Anthony W. D. Larkum, Anya Salih, Michael Kühl

**Affiliations:** 1 School of Biological Sciences, University of Sydney, Camperdown, New South Wales, Australia; 2 Confocal Bio-Imaging Facility, School of Natural Sciences, University of Western Sydney, Richmond, New South Wales, Australia; 3 Marine Biological Section, Department of Biology, University of Copenhagen, Helsingør, Denmark; 4 Plant Functional Biology and Climate Change Cluster, University of Technology, Sydney, Broadway, New South Wales, Australia; United States Department of Agriculture, Agricultural Research Service, United States of America

## Abstract

**Background:**

The calcifying siphonalean green alga, *Halimeda macroloba* is abundant on coral reefs and is important in the production of calcium carbonate sediments. The process by which new green segments are formed over-night is revealed here for the first time.

**Methodology/Principal Findings:**

Growth of new segments was visualised by epifluorescence and confocal microscopy and by pulse amplitude modulation (PAM) fluorimetry. Apical colourless proto-segments were initiated on day 1, and formed a loose network of non-calcified, non-septate filaments, containing no chloroplasts. Rapid greening was initiated at dusk by i) the mass movement of chloroplasts into these filaments from the parent segment and ii) the growth of new filaments containing chloroplasts. Greening was usually complete in 3–5 h and certainly before dawn on day 2 when the first signs of calcification were apparent. Mass chloroplast movement took place at a rate of ∼0.65 µm/s. Photosynthetic yield and rate remained low for a period of 1 to several hours, indicating that the chloroplasts were made *de novo*. Use of the inhibitors colchicine and cytochalasin *d* indicated that the movement process is dependent on both microtubules and microfilaments.

**Significance:**

This unusual process involves the mass movement of chloroplasts at a high rate into new segments during the night and rapid calcification on the following day and may be an adaptation to minimise the impact of herbivorous activity.

## Introduction

Calcifying siphonalean green algae in the genus *Halimeda* are abundant on coral reefs around the world [1] and are important in the sequestration of carbon from the atmosphere [1], [2]. *Halimeda* species typically produces segmented plants that are attached by a holdfast, which can be attached to rock or coral or buried in sediment. Bundles of the aragonite crystals from dead *Halimeda* plants are an abundant source for the fine-grained sediments on many coral reefs [1]. As in all members of the Order Siphonales, the thallus is formed from branching filaments, which have few septa and thus the plant is coenocytic. In *Halimeda*, the thallus has central filaments arranged in an axial orientation, giving rise to radial filaments in the cortex. The cortical radial filaments branch extensively and fuse at the apices of branches to form a closed surface [3]; beneath this closed surface there is free space between the filaments known as the inter-utricular space. Large numbers of aragonite crystals are found in the inter-utricular space. Chloroplasts occur mainly in the outer cortical filaments adjacent to the inter-utricular spaces. The coenocytic morphology renders the plant potentially vulnerable to damage and grazing, and plants in this Order possess very rapid wound responses and produce feeding deterrent chemicals [4], [5].

*Halimeda*-dominated communities have been found to be important primary producers in many regions of the world [2], [6], [7], [8], [9], [10]. On shallow reefs, they are significant contributors to soft sediments [1], and *Halimeda* species have been shown to produce massive carbonate deposits in deeper-water sites, both in the modern world [9], [10], [11] and in the geological past [12]. *Halimeda* is thus important both in geological sediment production [2], [11], [12] and in making a significant contribution to carbon sequestration [2].

A number of studies have been carried out on *Halimeda spp.* with a view to clarifying processes of photosynthesis, growth, calcification and chloroplast movement. Borowitztka and Larkum studied calcification and chloroplast formation [3], [13], [14], [15], [16], [17], [18]. They concluded that photosynthesis during the day causes alkalinisation of the inter-utricular space and that this is the trigger for calcification [3]. Subsequent microelectrode studies [19] supported this hypothesis, although it has not yet been possible to obtain *in situ* results for the inter-utricular space with pH microelectrodes because wound reactions lead to the release of acids around the microelectrode. While photosynthesis undoubtedly plays a key role, ion transport processes across the utricular filament membranes [3], [15] and nucleation sites in the inter-utricular space [20] are also important for the calcification process. Borowitzka & Larkum [13], [14] also studied chloroplast formation: they showed that new plastids formed from proplastids and that a ‘thylakoid organising body’ was an intermediate step in the maturation process [14].

Plastid movement in *Halimeda* species has been studied by Drew and coworkers. In the dark, chloroplasts are withdrawn inwards in the radial outer filaments [21], returning to the perimeter the following day just before dawn. The segments thus pale visibly in the dark, turning from a dark green colour to almost white. This is largely a response to light, since segments will also pale during the day if shaded, but there is also a regulatory component with an endogenous rhythm [22].

New segments of *Halimeda* plants are formed monthly [23]. In this process, a new colourless proto-segment is initiated during the course of the first day. This flaccid young segment is formed of a loose network of non-calcified, non-septate filaments, bathed in seawater and initially containing no chloroplasts. During the following night, chloroplasts move from the mature parent segment below into the developing segment. Apart from qualitative descriptions of this process, no detailed reports of the sequence of events or the possible mechanisms are available. Here we have used confocal microscopy and variable chlorophyll fluorescence imaging, with inhibitors and light manipulations, to study the process of the formation and greening of new segments in *Halimeda macroloba.*

## Materials and Methods

### Sampling

Plants of *Halimeda macroloba* Decaisne were collected on the reef flat adjacent to the Heron Island Research Station (152°06′E, 20°29′S) on the Great Barrier Reef, Australia. Plants were kept in running seawater tanks under shaded conditions at 23–25°C. For the confocal work, material was kept in a seawater aquarium at the University of Sydney over a period of 3 months at 25°C under white growth-light fluorescence tubes at an irradiance of ∼250 µmol photons m^−2^ s^−1^ during a 12 h day.

### Microscopy

Initial work was carried out using a compound microscope (BH2-RFL, Olympus, Japan) with a reflected light fluorescence attachment at 10X (DPLAPO10XUV, Olympus, Japan) and 40X (DAPO40UV/RiO, Olympus, Japan) magnification. The excitation light source was a 100W high-pressure mercury lamp (HBO 100W/2, Olympus, Japan). An excitation filter (UG-1, Olympus, Japan) was used in combination with a dichroic filter U-V (400–455 nm) for chlorophyll fluorescence (∼680 nm), and an excitation filter BP-545 in combination with a dichroic filter B–G for green (cell wall) fluorescence (500–580 nm).

### Confocal Microscopy

*H. macroloba* thallus segments were imaged in seawater mounted on a glass cover slip, which was fitted over a drilled glass slide. Typically, three regions of interest were imaged at different time intervals – the bottom, the middle and the growing edge of each sampled new segment. Confocal imaging was done on a Leica DMIRBE inverted microscope fitted with a TCS SPII confocal head (Leica Microsystems, Heidelberg, Germany) using 10X NA 0.35 dry, 40X PLAPO CS 0.75 oil or 63X HCX PLAPO CS water immersion objectives (Leica Microsystems, Heidelberg, Germany). We used the 488 nm excitation line of an Argon multi-line laser and the triple dichroic TD 488/543/633 nm beam splitter. Fluorescence emissions of cell walls and the meshwork of growing filaments were detected in photomultiplier (PMT) 1 at 500–530 nm and PMT 2 at 570–620 nm; chlorophyll in chloroplasts was imaged by fluorescence at 670–700 nm. Structural analysis was performed by 3-dimensional (3D) reconstruction of z-stacks containing serial sections (“xyz” scan mode) taken from each sample surface at 5 µm steps and to a depth of 40–120 µm. Complete 3D models of the specimen were rendered and examined at different orientations using Leica Microsystems software. Chloroplast streaming was imaged using the “xyt” time-scan mode. Spectral changes during segment growth and maturation were monitored at 488 nm or 514 nm laser line excitation and imaging was done by microspectral detection in “xyλ” mode at 500–700 nm or 520–700 nm, respectively, using a 500 RSP dichroic filter to block excitation light.

Cellular aragonite (calcite) deposition was imaged using the reflection mode of the confocal microscope with a violet 458 nm laser line (high light scattering) positioned on top of PMT 1 at 450–470 nm. Fluorescence emissions in green-yellow wavebands were imaged in PMT 2 and chlorophyll fluorescence emissions at 680–700 nm were detected in PMT3. 3D sections were collected at 2.28 µm increments to a depth of 200 µm. All imaging was done at a scan speed of 400 Hz and image size of 512×512 pixels.

### Variable chlorophyll fluorescence imaging

The distribution and photosynthetic activity of chloroplasts in *Halimeda* plants was mapped simultaneously with an imaging pulse-amplitude-modulated chlorophyll fluorimeter (Imaging-PAM, Walz GmbH, Germany)[24]. The system uses weak modulated blue light to probe the status of PSII by measuring the chlorophyll fluorescence yield in the dark-adapted state (F_0_) and during a strong saturation pulse (F_m_), which drives photosystem II into a closed state. By this saturation pulse method [24], [25] it is possible to determine the maximum quantum yield as:
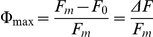
Similarly, the effective quantum yield of PSII (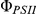) can be determined under a known actinic irradiance, using the fluorescence yield under ambient irradiance (F) and the fluorescence yield from a saturating pulse (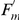)_,_ as follows: 
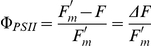
These quantum yields can be transformed to a relative measure of photosynthetic electron transport rate (ETR) as follows:
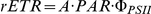
where the absorptivity, A, is either set to a constant or estimated from reflectance measurements. The imaging-PAM (i-PAM) system estimates A from imaging the reflectance of red (photosynthetically active) light (R) and near-infrared (photosynthetically inactive) light (R_NIR_) from a sample:
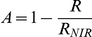
Many other parameters of photochemical and non-photochemical quenching can be determined by the saturation pulse method [24], [25]. In this study we took advantage of the ability of the i-PAM system to map i) the distribution of chlorophyll/chloroplasts via fluorescence yield measurements, ii) the absorption of red light, and iii) chloroplast activity via the quantum yield of PSII over several time points at intervals of 5–15 minutes.

### Oxygen microelectrode measurements

We recorded the O_2_ conditions in the proto-segment during its maturation with an O_2_ microelectrode with a tip diameter of 10 µm [26]. The microelectrode was connected to a pA-meter (PA2000, Unisense AS, Denmark) and measuring signals were acquired on a strip-chart recorder (SE110, ABB Goertz, Austria). The microelectrode was linearly calibrated from readings in aerated seawater and water deoxygenated with gaseous nitrogen. The microsensor was mounted in a manually operated micromanipulator (MM33, Märtzhäuser, Germany) and the measuring tip was carefully positioned into the center of the *Halimeda* proto-segment, which was kept in a small chamber with aerated seawater at a temperature of 25°C. We used a fiber-optic tungsten-halogen lamp with a collimating lens (KL2500, Schott GmbH, Germany) with the output fiber as a light source. The irradiance used in the morning after measuring the O_2_ depletion during darkness was ∼365 µmol photon m^−2^ s^−1^.

### Inhibitors and statistical analysis

Inhibitors were obtained from Sigma Chemical Co. A standard “R” statistics package was use for the chi-square analysis.

## Results

### General Observations and Microscopy

**Figure 1:**
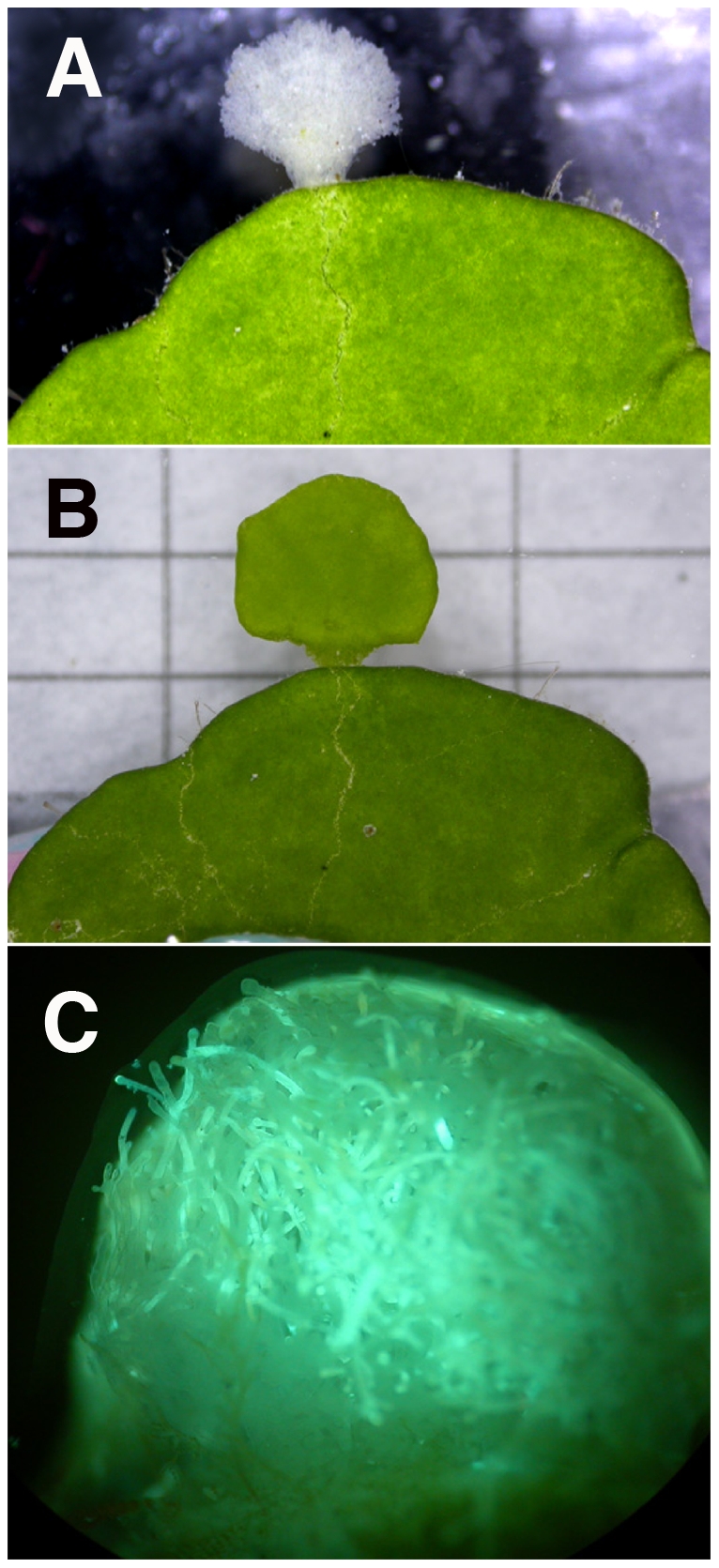
Morphology of proto-segments and greened young segments of *Halimeda macroloba*. Photographs of A) a white proto-segment of *H. macroloba*, B) the same segment greened 14 h later, and C) epifluorescence microscopy image of a young, non-green proto-segment as in (A), showing surface scaffold filaments.

Observations in the field of >200 plants and over two summers indicated that white to yellowish proto-segments were formed during daylight hours (day 1) (Fig. 1A–B). Ten plants with proto-segments were collected in the late afternoon and were kept under ambient light in flowing seawater at 25°C. The proto-segment is formed of large-diameter (40–80 µm) poorly branched ramuli, called scaffold filaments in the following. There appears to be a general synchronicity in the process of proto-segment formation with most plants showing new growth at the same time, i.e. the same day of the month, once a month. However, on any given day, apart from the major event, about 10% of plants showed one or more new segments on one or more branches of the thallus.

**Figure 2:**
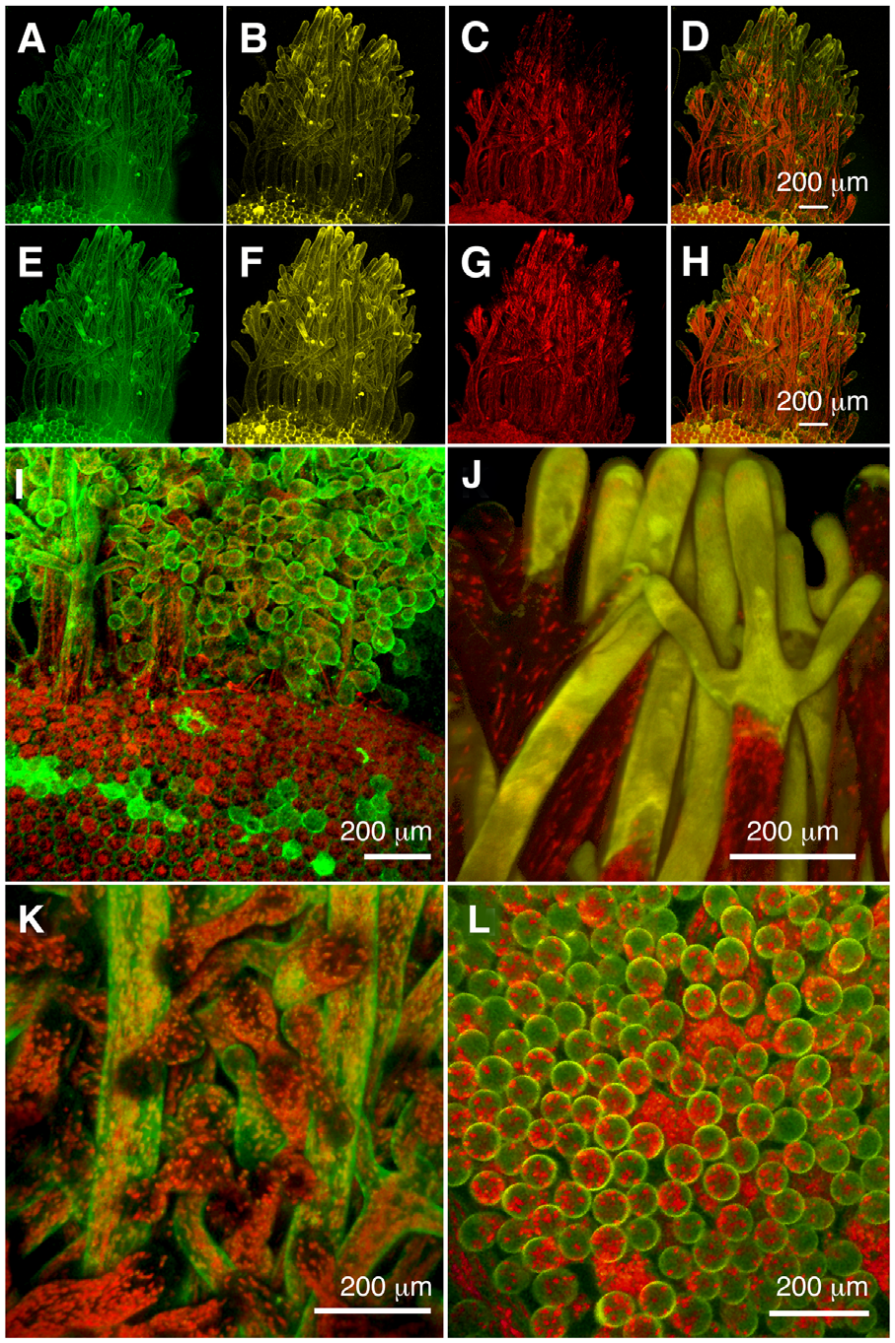
Fine-structural details of greening of proto-segments: confocal images of a proto-segment in *H. macroloba* viewed as 3D reconstructions. Newly formed proto-segment imaged under 488 nm excitation showing (A) mainly green emissions, (B) weak yellow emissions from filaments, that are brightest at the tips, as well as (C) red Chl emissions from chloroplasts, mainly absent from tips. (D) Superimposed image of a–c. The same proto-segment imaged after ∼1 h, showing increased fluorescence of filaments with (E) strong green and (F) yellow emissions, concentrated at filament tips, and (G) increased numbers of plastids spreading to filament tips. (H) Superimposed image of a–c. (I) The edge of parent segment and the base of the proto-segment, with green and yellow emissions imaged in a single channel, showing increased number of filament branches with time. (J) The tips of filaments become strongly fluorescent due to yellow-orange emissions of the cytoplasm and cell walls. (K) Filament side-branches become intertwined, forming the scaffold filaments. (L) Confocal image of the middle region of a developing proto-segment after 14 h, in which filament side-branches turn outwards; just before they fuse, the filaments form a hexagonal cell surface view common to all parent segments, as seen in lower part of I.

Epifluorescence microscopy showed strong green fluorescence of the cell walls of filaments in newly formed proto-segments (Fig. 1C; 2A); after 1–2 hours, this fluorescence gradually increased and changed from green to bright yellow-orange emission (compare Fig 2A–D vs E–H). Confocal microspectroscopic analysis of filament tips showed peak emissions at 540 and 583 nm, with 488 and 514 nm excitation, respectively. The yellow-orange fluorescence was especially prominent and was present in cell walls and the cytoplasm (Fig. 2J).

After dusk on the first day (ca 18.30 h in summer), greening of proto-segments occurred as a result of two processes: i) phase 1, movement of plastids in the cytoplasm inside the preformed scaffold filaments, originating from the parent segment, and then into smaller branches (Fig. 2A–H), and ii) phase 2, production of new green filaments by the parent segment, which grew into the proto-segments, intertwining the scaffold filaments (Fig. 2I, K).

#### Phase 1

**Figure 3:**
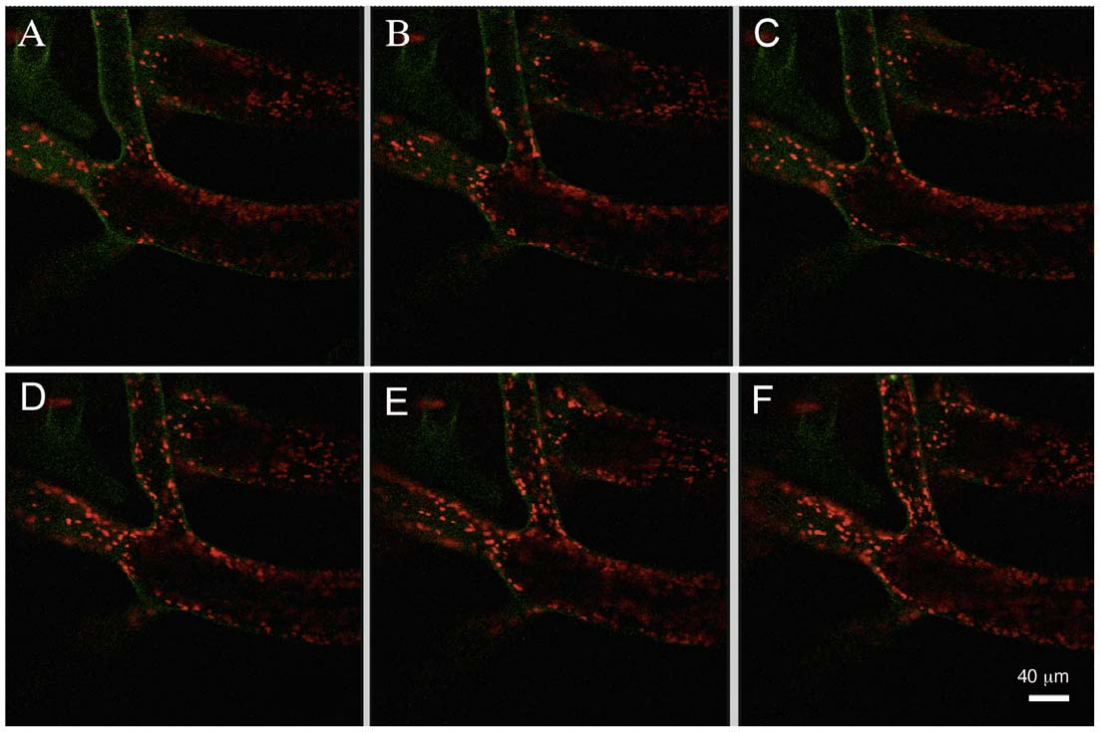
Movement of chloroplasts into a scaffold filament of a proto-segment of *Halimeda macroloba*. Confocal images were taken at intervals of 1.7 min (A–F) showing movement of chloroplasts near the base of a young proto-segment soon after chloroplasts had begun to flow into the filament.

In the first process, the rate of movement of chloroplasts was observed by confocal “xyt” imaging. Plastids moved by cytoplasmic streaming, with plastids being carried randomly; some individual chloroplasts moved in the reverse direction, but there was a net movement of chloroplasts in the distal direction (see Fig. 3). In 56 randomly selected plastids, in 14 separate filaments, we obtained a rate of 0.48 (±0.24) µm s^−1^ (mean ± SD; n = 56). The greening process was observed visually many times on Heron Island with freshly collected material as well as in the laboratory with confocal microscopy, over 6 non-consecutive nights using 5 plants kept in the aquarium under normal (dark conditions) and 5 plants kept illuminated over night. Much binary fission of chloroplasts was observed under high power microscopy (x1200).

#### Phase 2

**Figure 4:**
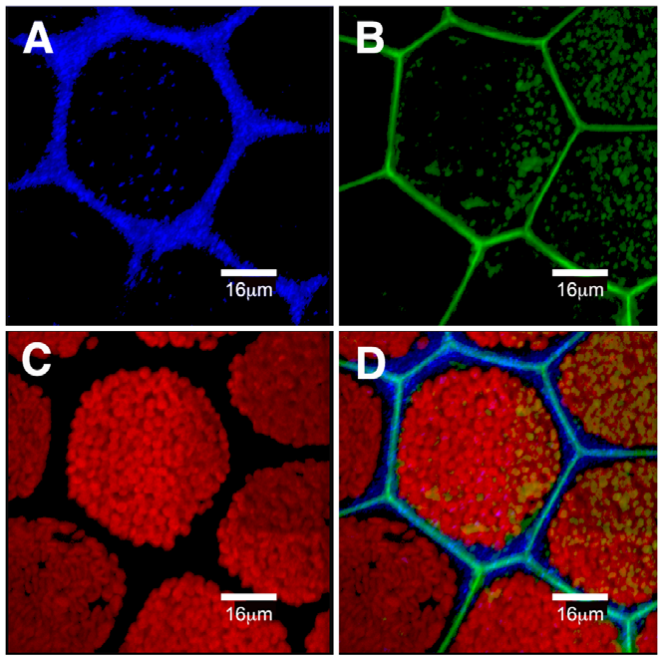
Surface view of maturing proto-segment in *Halimeda macroloba* showing the outer face of a utricular filament, in which the tips have fused and deposition of aragonite crystals is occurring. The confocal images represent a 3D view of serial images taken at 0.2 mm increments under 458 nm excitation. (A) Reflection mode showing newly formed aragonite crystals in the intra-utricular spaces imaged at 450–470 nm. (B) In fluorescence mode showing cell wall emission at 510–590 nm, where it is fused to adjacent utricular filaments. (C) Chlorophyll emission from chloroplasts at 680–700 nm. (D) Composite of A), B) and C).

The initial rapid movement of chloroplasts into the scaffold filaments was followed by ramification of the initial ramuli, which formed many sub-branches, and by the growth of new ramuli from the parent segment. By dawn on day 2 (ca 05.30 h in summer), the secondary filaments had penetrated throughout the proto-segment and projected further in all directions, but particularly perpendicular to the surface, where many small branchlets were formed that became young utricles. These young utricles could be seen packed closely together from dawn on day 2 (Fig. 2I) and thereafter began to fuse forming a closed, hexagonal surface identical to the surface of mature segments (Fig. 2L). During the morning of day 2, calcium carbonate (aragonite) crystals [3] were observed in the inter-utricular spaces, close to the cell walls of the young utricles (Fig. 4); aragonite crystals are highly light-scattering and their appearance within cells was visualized by using confocal microscopy in reflection mode (see Materials and Methods).

This developmental pattern was typical for most plants; however, in a few plants (<10%) it took most of the night to complete the growth of green filaments. Such slow growth was particularly noted in young plants with thick mature segments and few branches; in one such case the new segments (8 in all) were still white the following morning and greening occurred only over the course of 5 h in daylight on day 2.

In all cases, growth continued on day 3 and by day 4 the new segments approached the size of mature segments. In the aquarium-grown material, segment formation did not appear to follow any periodicity and occurred randomly in plants from day to day, possibly as a result of disruption of their natural diurnal light cycles. Nevertheless, it was still possible to identify proto-segments on the afternoon of day 1 and to then observe greening during the night and calcification on the following day.

In an experiment with 10 plants under illumination (∼200 µmol photon m^−2^ s^−1^, white light) from 16.00 h on day 1 until 07.00 h on day 2 no green filaments were produced during the night. After transfer to natural light, green filaments began to grow in the early morning (ca 8.00 h, 2.5 h after dawn) of day 2, and by the afternoon of day 2 they were similar to filaments on plants darkened overnight. Plants (n = 5) collected after 17.00 h on day 1, but illuminated overnight, as before, tended to have new segments that greened overnight, although the timing was delayed. Plants (n = 5) irradiated with red light (∼100 µmol photon m^−2^ s^−1^, light >600 nm) overnight all had white proto-segments on day 2 and greened up (in daylight) on day 2.

### Imaging-PAM fluorometry

**Figure 5:**
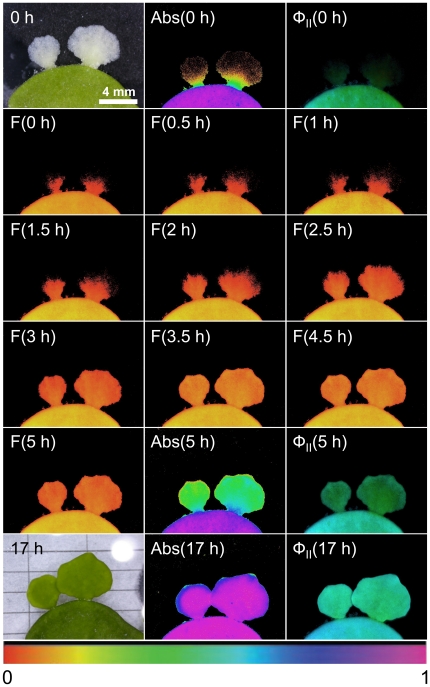
Sequence of new segment development and the onset of photosynthetic activity in *Halimeda macroloba.* The imageds wer eobtained by digital photography, absorptivity (Abs), fluorescence yield (F) and quantum yield of PSII (Φ_PSII_) over a 17 h period using microscopy and imaging-PAM fluorimetry. An animation of the full dataset, i.e., images taken every 5–10 minutes is available in Supporting Information: Movie S1. All colour images were normalised to the same relative colour code shown in the lower part of the figure.

On 4 nights using field material, a sub-sample of the normal (dark) treatment was taken for imaging PAM fluorimetric analysis. This necessitated subjecting the plants to low modulated blue light pulses and short (<1 s) saturating blue light pulses every 5–15 min. A typical dataset is shown in Fig. 5 for one such treatment (and an animation is shown in Supporting Information: Movie S1). At time zero, the white proto-segment exhibited weak chlorophyll fluorescence and absorptivity and low quantum yield confined to the area closest to the old segment. Over the course of 5–6 hours, the new green segment was formed and chloroplasts migrated into its filaments. The greening of the segment was largely completed after ∼3–5 h, while active photosynthesis was first detected after 5 hours. In the following 12 hours, the segment calcified and gained volume, while the maximum quantum yield was largely unchanged.

Usually, chloroplasts had reached the tips of the scaffold filaments after 180 min and the process of chloroplast movement was complete in ∼210 min (Fig. 5). The chloroplasts moved at a fairly constant rate, as evidenced by the rate of increase and spread of F_t_ throughout the new segment. Based on a mean base-to-tip length for the proto-segment of 7.0±0.1 mm (mean ± SD; n = 10) and a greening rate of 3 h, this would represent a chloroplast movement rate of 0.65 µm s^−1^, which is similar to rates observed directly under confocal imaging (unpublished data), but somewhat slower than the more random movent observed above for individual chloroplasts. Initially the areas with chlorophyll fluorescence showed little or no quantum yield, i.e., they were unable to carry out photosynthesis. The capacity for photosynthesis lagged ∼1 h behind completion of the chloroplast migration (see Fig. 5), as did the ability to carry out non-photochemical quenching (NPQ)(not shown).

### Oxygen concentration in the proto-segment

Microelectrode measurements showed O_2_ depletion (0–5% air saturation) in the center of the proto-segment throughout the dark incubation period where the maturation of the new segment occurred (data not shown). When actinic light was provided in the morning after the greening process had completed, the O_2_ quickly rose to levels exceeding those in the surrounding seawater kept at 100% atmospheric saturation. These measurements thus showed a pronounced O_2_ respiration in the proto-segment during maturation and fully competent photosynthesis only upon completion of the maturation process.

### Inhibitor studies

Table 1 shows the results of the experiment with colchicine (0.5 mM) and cytochalasin *d* (2 µM), with 20 replicates. Alone, each inhibitor partially inhibited greening. However, with both inhibitors present together there was a total inhibition of new filament growth, where the proto-segments (n = 20) were approximately the same size on the morning of the second day, as at dusk the previous day, and without formation of utricle initials. Cytoplasmic streaming and filament growth were only fully inhibited in the presence of both inhibitors, while partial inhibition was induced when only one of the inhibitors was applied. Although the numbers are small, results are clearly significant and this was confirmed by using Pearson's chi-square test on the complete set of data (p<2.2e^−16^).

**Table 1:** The effect of the inhibitors colchicine (0.5 mM in seawater) and cytochalasin *c* (2 µM in seawater), on the greening of proto-segments in *Halimeda macroloba* (see text for further description).

Treatment	Replicates	Colour after 12 h (in darkness)		
		White	Partially green	Fully green
Control	20	0	0	20
Colchicine (0.5 mM)	20	2	16	2
Cytochalasin *d* (2 µM)	20	4	16	0
Colchicine + Cytochalasin *d*	20	20	0	0

## Discussion

The siphonalean green alga *Halimeda* has been studied for many years because of its special features in terms of calcification [3], [15], [16], [17], [18] and circadian chloroplast movement [21], [22]. The mechanisms involved in new segment formation have hitherto been unknown and our study shows for the first time that segment formation involves a complex series of events, whose further study could be a very valuable tool in plant cell research.

Once the proto-segment is formed, greening begins at dusk. Maturation is a three-part process, depending firstly on the import of green chloroplasts and proplastids into the scaffold filaments from the parent segment, together with replication of those plastids and secondly on the formation of new green filaments, which grow a) by ramification of the existing ramuli, and movement of chloroplasts in the large ramuli (Fig. 3) and b) by new filaments growing from the parent segment that interpenetrate the scaffold filaments of the proto-segment (Fig. 2), taking chloroplasts generated in the parent segment and proplastids. The incapacity of freshly imported plastids to perform full photosynthesis initially (Fig. 5) is an interesting observation, suggesting that these are newly formed organelles, whose maturation occurs during the night. As the new segment greens overnight maturation affects several photosynthetic fluorescence parameters (Fig. 5), most notably Fm, Fm' and NPQ, suggesting that proplastids mature into fully functional plastids during this process. The third stage of the maturation process is sealing off the inter-utricular space from the outside, the formation of aragonite crystals (Fig 4) and the evolution of photosynthetic oxygen on the morning of the second day. Following this, new segments continued to grow over the third and fourth day.

The rapid production of new plastids, over a typical time of 2–3 h, represents one of the fastest recorded maturations of photosynthetic tissue and therefore deserves closer scrutiny in future studies. The chloroplast movement into the initially colourless filaments via the scaffold filaments (Figs. 2, 3 & 5) occurred at a fast rate of ∼0.65 µm s^−1^. Ten fold higher intracellular transport rates have been observed for cytoplasmic streaming in *Caulerpa prolifera,* a member of the Siphonales, while in *Nitella,* a streptophyte alga, an hundred fold higher rate was recorded [27]. However, these higher rates were not for plastid movement, nor were they a mass unidirectional movement.

The chloroplast movement in *H. macroloba* could only be completely inhibited by the simultaneous presence of the inhibitors colchine and cytochalasin *c* (Table 1), which inhibit microtubule- and microfilament-dependent cell organelle movement, respectively [28]. Thus both types of cellular transport mechanisms are involved in chloroplast movement of *H. macroloba*. The formation and transport of chloroplasts is, however, only one aspect of the remarkable growth spurt in *H. macroloba* and, for example, the rate of protein synthesis must also be very high. The proto-segment interior became anoxic during night-time maturation indicating a high respiratory load during this period, presumably as a result of the high demand for ATP. Thus this system should provide a useful tool for future studies of protein synthesis and growth in algae.

The temporal triggering of new segment formation in *H. macroloba* is intriguing as it apparently involves a periodicity in the majority of plants, which may, for example, be entrained to a lunar cycle, as has been found for other green macroalgae [29]. However, about 10% of plants do not follow this cycle. Once the proto-segment is initiated, the timing of the greening process seems dependent on a light/dark sensor system, whereby growth is initiated at dusk on day 1. Continuous light overnight could delay development until the next morning when inhibition was alleviated and growth proceeded. Our finding that red light had a similar effect to white light in causing such a delay could indicate that a phytochrome system is involved.

The proto-segment is formed of scaffold filaments recruiting chloroplasts and becoming intertwined by secondary green filaments from the parent segment at night-time. We hypothesize that grazers attempting to feed on the newly arising structure during daylight of day 1 would be deterred by the tough nature of the proto-segment filaments, lack of nutrition and the possible presence of secondary metabolites. The walls of the proto-segment filaments are thickened and the fluorescent nature of the walls suggests that tannins or other phenolics may be present as a feeding deterrent. It is known that *Halimeda* spp. produce diterpenoid feeding deterrents [4], [5], [30] and such compounds may also be present in the proto-segment.

The end result of the greening process is a mature segment with protection from grazing by the presence of secondary metabolites and the production of unpalatable aragonite crystals (Fig 4). Borowitzka & Larkum [3] suggested that a major factor in the calcification process was the alkalinisation of the inter-utricular compartment, as a result of photosynthesis in adjacent chloroplast-packed utricular filaments in the light. This hypothesis was supported by the later work of Borowitzka [18] and De Beer & Larkum [19]. Unfortunately, it was not possible in the latter study to place a microsensor in the inter-utricular space owing to wound reactions. In mature segments, at night, chloroplasts are withdrawn deeper into the thallus and out of the inter-utricular filaments [21], [22]; thus illumination at night should not result in alkalinisation and calcification.

The reason(s) for the complex set of events leading to the generation of a new green segment in *H. macroloba* is(are) still a matter of conjecture. The phenomenon and its timing may be explained as a process minimizing herbivory. Herbivorous fish and molluscs would potentially be attractive to young, green segments. However mature segments of *Halimeda* species, even at the ends of branches, are tough, packed with aragonite crystals and filled with diterpenoid feeding deterrents [4], [5], [30], which are avoided by many reef herbivores. Although white proto-segments would be easy to ingest, they would be relatively unattractive, low in food value and, furthermore, may be replete with feeding deterrents. Our observations suggest that proto-segments are not actively grazed at night-time when there are few active herbivores, and this is when the chloroplasts are imported into the proto-segment, and maturation occurs. By first light on the following day, the utricles generally have been fused, whereafter calcification quickly fills the new green segment with aragonite crystals, increasing its indigestibility.

The hypothesis of herbivory avoidance could be tested by identifying, for example, when secondary metabolites are imported into the proto-segments and by assessing the palatability of proto-segments and new green segments to herbivorous fish and other grazers. It should be noted, however, that Mantyka & Bellwood [31] have shown that many macroalgae on coral reefs, including several species of *Halimeda,* are susceptible to heavy grazing by reef fish, when more attractive algal species are not available.

In summary, the white proto-segment of *Halimeda* is a novel structure that has received little attention in the past. We present the first detailed study of the rapid structural changes and cellular transport processes involved in segment formation and maturation. The fast movement of *H. macroloba* chloroplasts, inactive in photosynthesis, into scaffold filaments seems to represent a new phenomenon of mass migration of plastids not previously reported. The whole system provides a fascinating field of research for future studies in plant cell biology

## Supporting Information

Movie S1 An animation showing the greening of segments of *Halimeda macroloba* over 6 hours as imaged by an imaging-PAM (conditions were the same as for Fig. 5).
(WMV)
